# Graphene-modified CePO4 nanorods effectively treat breast cancer-induced bone metastases and regulate macrophage polarization to improve osteo-inductive ability

**DOI:** 10.1186/s12951-020-00753-9

**Published:** 2021-01-07

**Authors:** Yu-Wei Ge, Xiao-Liang Liu, De-gang Yu, Zhen-An Zhu, Qin-Fei Ke, Yuan-Qing Mao, Ya-Ping Guo, Jing-Wei Zhang

**Affiliations:** 1 Shanghai Key Laboratory of Orthopedic Implants, Department of Orthopedic Surgery, Shanghai Ninth People’s Hospital, Shanghai JiaoTong University School of Medicine, Shanghai, 200011 China; 2 The Education Ministry Key Lab of Resource Chemistry and Shanghai Key Laboratory of Rare Earth Functional Materials, Shanghai Normal University, Shanghai, 200234 China

**Keywords:** Scaffolds, Polarization, Graphene oxide, Bone regeneration, Near-infr

## Abstract

**Background:**

Breast cancer bone metastasis has become one of the most common complications; however, it may cause cancer recurrence and bone nonunion, as well as local bone defects.

**Methods:**

Herein, In vitro, we verified the effect of bioscaffold materials on cell proliferation and apoptosis through a CCK8 trial, staining of live/dead cells, and flow cytometry. We used immunofluorescence technology and flow cytometry to verify whether bioscaffold materials regulate macrophage polarization, and we used ALP staining, alizarin red staining and PCR to verify whether bioscaffold material promotes bone regeneration. In vivo, we once again studied the effect of bioscaffold materials on tumors by measuring tumor volume in mice, Tunel staining, and caspase-3 immunofluorescence. We also constructed a mouse skull ultimate defect model to verify the effect on bone regeneration.

**Results:**

Graphene oxide (GO) nanoparticles, hydrated CePO_4_ nanorods and bioactive chitosan (CS) are combined to form a bioactive multifunctional CePO_4_/CS/GO scaffold, with characteristics such as photothermal therapy to kill tumors, macrophage polarization to promote blood vessel formation, and induction of bone formation. CePO_4_/CS/GO scaffold activates the caspase-3 proteasein local tumor cells, thereby lysing the DNA between nucleosomes and causing apoptosis. On the one hand, the as-released Ce^3+^ ions promote M2 polarization of macrophages, which secretes vascular endothelial growth factor (VEGF) and Arginase-1 (Arg-1), which promotes angiogenesis. On the other hand, the as-released Ce^3+^ ions also activated the BMP-2/Smad signaling pathway which facilitated bone tissue regeneration.

**Conclusion:**

The multifunctional CePO_4_/CS/GO scaffolds may become a promising platform for therapy of breast cancer bone metastases. 
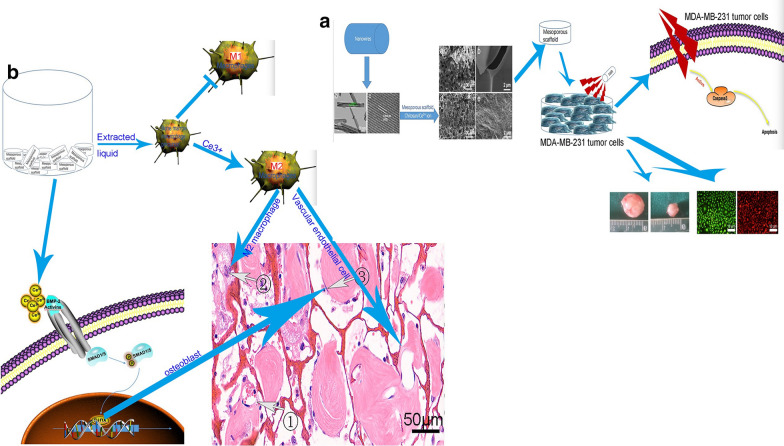

## Background

Breast cancer is a common form of malignant tumor in females [1–3]. In recent years, more cases of bone metastasis of breast cancer have been seen with an increasing incidence of this malignant tumour tumor [4]. Bone metastasis is the most common distant metastasis of breast cancer [5–7]. With bone metastasis, pathologic fractures can easily occur, which lead to symptoms such as pain, bleeding, and neurological dysfunctions [8, 9]. Furthermore, the combination of surgical removal and radiotherapy/chemotherapy is typically the main treatment method for bone metastasis caused by breast cancer [10–13]. However, a small fraction of the lesion can remain at the excision site after surgery, thus increasing the risk for recurrence of malignant tumors [14]. The physical condition of the patient is often poor after radiotherapy or chemotherapy, which can cause adverse effects on the fusion between bones and prostheses in the surgical region and even complications such as non-union [15, 16]. Therefore, there is a pressing need for a scaffold for tissue engineering that can improve the micro-environment for simultaneous tumour therapy and bone defect repair.

As compared with radiotherapy and chemotherapy, photothermal therapy (PTT) attracted increasing attention because of its highly efficient antitumor effect, precise spatial–temporal selectivity and no harm to healthy tissues and organs [17–19]. A variety of photothermal agents, such as copper-palladium alloys [19], graphene [20], carbon dots [21], magnetic nanoparticles [22] and Cu7.2S4 Nanoparticles [23] were developed for tumour therapy. The near-infrared (NIR) laser irradiation significantly elevates the local temperatures around photothermal agents to kill cancer cells via hyperthermia, and inhibit the expression of the metastasis-related factors such as matrix metalloproteinase, twist and transforming growth factor-β1 [24]. Notably, graphene oxide (GO) with excellent light‐absorbing property and photothermal conversion efficiency has been widely used for PTT application [20, 25]. Moreover, the carbonyl, epoxy and hydroxyl groups in the GO can serve as bioactive sites for cell proliferation and bone mineralization via activating MAPK signaling pathway [26–28]. It is reasonably inferred that the GO-modified scaffolds are fit for PTT against tumors and subsequent bone defect healing.

To effectively repair bone defects duo to tumour resection, bone biomaterials should provide appropriate media for angiogenesis and osteogenesis [29]. In vitro, M2 macrophages directly regulate osteogenic differentiation of bone marrow mesenchymal stem cells [30, 31]. The mechanism may be due to pro-regenerative cytokines produced by M2 macrophages, such as TGF-β, VEGF, Arg-1 and IFG-1 [32]. Angiogenesis is a critical step in bone regeneration and requires the provision of adequate nutrients, oxygen and circulating progenitor cells to bone tissue. M2 macrophages secrete VEGF. The VEGF might activate MMP-9, which can induce migration and proliferation of angioblast; Arg-1, as one of the most effective growth factors involved in angioblast recruitment. The scaffold can inhibit the aggregation of pro-inflammatory macrophages and the formation of fibrous connective tissue between the bone tissue and prosthesis, which promotes the fusion of the scaffold material and osteoblasts [20, 21].

Rare earth elements (REEs) accumulate in human bones, and easily substitute part of the Ca^2+^ ions in apatite nanoparticles because of their similar ionic radius [33]. In the safety concentration ranges, the element cerium (Ce) is employed as a therapeutic agent to induce bone tissue growth via enhanced angiogenic and osteogenic activities [34, 35]. Cerium oxide nanoparticles in scaffolds activate calcium channel of mesenchymal stem cells (MSCs) and up-regulate vascular endothelial growth factor (VEGF) expression, resulting in enhanced vascularization [33]. Furthermore, the as-released Ce^3+^ ions from bone grafts activate the SMAD signaling pathway which improves the differentiation of MSCs into osteoblast [35]. However, the high concentrations of Ce may cause toxicity to healthy bone tissues. In order to control the Ce^3+^ concentrations in vivo, CePO_4_ nanorods with a high crystallinity are desired candidates because of their low degradability. In addition, chitosan (CS) as a biocompatible macromolecule, has been widely used for bone repair materials [34]. It is reasonably inferred that the incorporation of CePO_4_ nanorods in CS matrix may become a promising-biomaterials to repair bone defects.

For the postoperative therapy of breast cancer-induced bone metastases, we developed multifunctional CePO_4_/CS/GO porous scaffolds by a freeze-drying strategy. We hypothesized that the GO nanosheets in the scaffolds could enhance photothermal conversion efficiency for the PTT of residual tumor cells after surgery, and the CePO_4_ nanorods improve the vasculogenic and osteogenic activities for the subsequent bone defect repair. In order to prove this hypothesis, we performed in vitro and in vivo tests to demonstrate the therapeutic efficacy of multifunctional CePO_4_/CS/GO scaffolds against breast cancer-induced bone metastases.

## Methods

### Preparation of CePO_4_ nanorods

1.3539 g Ce (NO_3_)_3_·6H_2_O and 0.0990 g (NH_4_)_2_HPO_4_ were dissolved separately in 30 ml ultrapure water. The Ce (NO_3_)_3_ solution was added dropwise into the (NH_4_)_2_HPO_4_ solution under continuous stirring. During the dropping process, 1.0 M of HCl was utilized to keep the pH value at 0.5. After further stirring for 30 min, and the mixtures were transferred to the autoclave. The hydrothermal reaction was carried out at 180 °C for 24 h, and then was cooled to a room temperature. The final products were washed with distilled water and alcohol, and dried at 55 °C.

### Preparation of CePO_4_/CS/GO scaffolds

1.00 g CS was added into 25 ml CH_3_COOH solution, forming a CS solution after mechanical agitation for 2 h. 2.00 g CePO_4_ nanorods and 0.09 g GO nanosheets were added to the CS solution. The mixed solution was stirred for 2 h, and then was transfused to the mould of 24-well or 96-well plates. The samples were freeze-dried in a freezer at -60ºC for 48 h. The CePO_4_/CS/GO scaffolds were washed by deionized water for 6 days, and freez-dried again. Furthermore, CePO_4_/CS scaffolds were constructed by the same method without adding GO nanosheets.

### Characterization

The morphology and crystal structure of CePO_4_ nanorods was detected by transmission electron microscopy (TEM, JEOL2100, Japan) and selected area electron diffraction (SAED). Field-emission scanning electron microscopy (SEM, JSM-6380LV, Japan) and energy-dispersive spectrometry (EDS) were performed to observe the morphologies and element distributions of the nanorods and scaffolds. The phases of the samples were assayed by an X-ray diffractometer (XRD; D/max-III C, Japan) in a 2θ range of 10–70° with Cu Kα radiation. Functional groups in samples were detected by Fourier transform infrared spectroscopy (FTIR; PerkinElmer, USA) in the wavenumber range of 4000–500 cm^−1^. The binding energies of the Ce in the CePO_4_ nanorods were detected by an X-ray photoelectron spectroscopy (XPS, PHI5700 ESCA, USA). The light adsorption properties of the scaffolds were detected by a UV–vis spectrometer (UV3600, Shimadzu) in 400–1200 nm region. The Ce ion release performance from CePO_4_/CS porous scaffolds was investigated by soaking 0.175 g CePO_4_/CS/GO scaffolds in 4.5 ml ultrapure water. After released for different time, the concentrations of Ce^3+^ ions were determined by inductively coupled plasma/optical emission spectrometry (ICP/OES; Perkin Elmer, OPTIMA 3300 DV). To determine photothermal effects of the scaffolds, 7.80 mg samples were immersed in 100 µL ultrapure water. The temperatures were detected with time by a thermocouple thermometer upon the irradiation of NIR laser (λ = 808 nm, 4.6 W/cm^2^).

### Cell culture and toxicity

MC3T3-E1, RAW264.7, and MDA-MB-231 cells were purchased from the Shanghai Institutes for Biological Sciences of the Chinese Academy of Sciences (China). Human bone marrow mesenchymal stem cells (hBMSCs) were purchased from the Shanghai Bio-Chain Biological Technology Co., Ltd. (China). All processes were approved by the Animal Hospital of Shanghai Jiao Tong University. The α-minimum essential medium (α-MEM) and foetal bovine serum (FBS) were purchased from the Gibco line of Thermo Fisher Scientific, Inc. (Waltham, MA, USA). Cells were cultured at 37 °C with 5% CO2. To examine the cell toxicity, 1 × 10^4^ MC3T3-E1 cells/well were seeded in a 96-well plate and cultured for 24 h. The extraction solutions of CS, CePO_4_/CS, and CePO_4_/CS/GO scaffolds were used as the exchange solutions. The cell toxicity was examined with Cell Counting Kit-8 (CCK-8 Dojindo, Kumamoto, Japan) on days 1, 2, 3, and 4.

MDA-MB-21 cells (metastatic breast cancer cells) were cultured in Dulbecco's modified Eagle's medium (DMEM) containing 10% FBS at 37 °C with 5% CO2. The cells were then mixed and cultured with the CS, CePO_4_/CS, and CePO_4_/CS/GO scaffolds (12 mm diameter, 2 mm height) in 24-well plates for 24 h under NIR radiation with a power density of 4.6 W cm^ − 2^ for 3 min. The cell viability was examined with Cell Counting Kit-8 (CCK-8 Dojindo, Kumamoto, Japan) on days 1, 2, 3, and 4.

### Alkaline phosphatase staining and alizarin red staining

MC3T3-E1 cells were cultured in a 24-well plate for 24 h. The CS, CePO_4_/CS, and CePO_4_/CS/GO extraction solutions were used as exchange solutions to test for cell osteogenesis. Cells were cultured in the extraction solutions for 7 and 21 days and fixed with paraformaldehyde (PFA), followed by two rinses with phosphate-buffered saline (PBS). The cells were then stained using an alkaline phosphatase (ALP) kit and alizarin red staining kit (Hongqiao, Shanghai, China) and observed under an optical microscope.

### Cell adhesion

The hBMSCs were cultured in 24-well plates at a density of 1 × 10^4^ cells/well with CS, CePO_4_/CS, and CePO_4_/CS/GO for 12 h. The cells were fixed with 2.5% glutaraldehyde for 20 min and then dehydrated in ethanol with concentration gradients of 75, 85, 95, and 100% before being observed under scanning electron microscopy (SEM, Siriaon 200, FEI, Hillsboro, OR, USA).

### Flow cytometry

The CS, CePO_4_/CS, and CePO_4_/CS/GO scaffolds were added to MDA-MB-231 cell culture medium, and the cells were exposed to radiation from a near-infrared (IR) spectrometer with a power density of 4.6 W cm^−2^ for 3 min. The apoptotic cells were detected using flow cytometry (annexin V/PI staining) according to the protocol provided by the manufacturer (BD Bioscience, USA). The RAW264.7 cells were then added to the CS, CePO_4_/CS, and CePO_4_/CS/GO scaffolds. The cells with the CePO_4_/CS/GO scaffolds were divided into two groups; one group was exposed to NIR radiation, and other was not. According to the protocol provided by the manufacturer (BD Bioscience, USA), the polarization of the macrophages was examined by flow cytometry using anti-mouse CD16/32-PE (cat. No. 553145) and anti-mouse CD206-Alexa 647 (cat. No. 565250).

### Immunofluorescence

The CS, CePO_4_/CS, and CePO_4_/CS/GO scaffold materials were added to other batches of RAW264.7 cell culture medium. The cells with CePO_4_/CS/GO were divided into two groups; one group was exposed to NIR radiation, and the other was not. The cells were fixed with 4% PFA fixation solution for 20 min. The CD206 (1:200) antibody and CD16/32 (1:200) antibody were employed for the immunofluorescence analysis. All antibodies were purchased from Cell Signaling Technology.

### Quantitative reverse transcription polymerase chain reaction (RT-qPCR) and western blot

The MC3T3-E1 cells were seeded at a concentration of 4 × 10^6^ in extraction solutions of CS, CePO_4_/CS, and CePO_4_/CS/GO scaffolds. The total RNAs were collected with an RNeasy Mini kit (Qiagen: Valencia, CA, USA) after 5 days of culture. Reverse transcriptase (TaKaRa) was employed to transcribe the RNA to complementary DNA (cDNA). SYBR1 Premix ExTaqTM II (TaKaRa) was added to the Real-Time PCR and ABI 7500 Sequencing Detection System (AppliedBiosystems, Foster City, CA). The PCR primer was designed as follows:

GAPDH forward 5′-CACCACCATGGAGAAGGCCG-3'

and reverse 5′-ATGATGTTCTGGGCAGCCCC-3'

Runx2 forward 5′-GACTGTGGTTACCGTCATGGC-3'

and reverse 5′-ACTTGGTTTTTCATAACAGCGGA-3'

ALP forward 5′-AGAAGTTCGCTATCTGCCTTGCCT-3'

and reverse 5′-TGGCCAAAGGGCAATAACTAGGGA-3'

BMP-2 forward 5′-CCGCTCCACAAACGAGAAAA-3′

and reverse 5′-CAGCAAGGGGAAAAGGACAC-3′

OCN forward 5′-TAGCAGACACCATGAGGACCATCT-3'

and reverse 5′-CCTGCTTGGACATGAAGGCTTTGT-3'

The expression levels of COL1, bone morphogenetic protein (BMP-2), P-Smad1/5, RUNX2, and GAPDH were examined with CS, CePO_4_/CS, and CePO_4_/CS/GO scaffold extraction solutions. After 3 days of culture, radioimmunoprecipitation assay (RIPA) lysate containing protease inhibitor was added to the MC3T3-E1 cells, and they were left for 20 min. The cells were centrifuged at 12,000 rpm for 20 min, and the supernatant was collected. The protein content of the supernatant was analysed by a bicinchoninic acid assay (BCA). The supernatant was transferred to gel pores for sodium dodecyl sulfate–polyacrylamide gel electrophoresis (SDS-PAGE) and then transferred to a poly (vinylidene fluoride) (PVDF) membrane after electrophoresis. The membrane was stored in 5% low-fat milk for 1 h. Primary antibody was added, and the samples were incubated at room temperature for 4 h. GAPDH(1:1000), P-Smad1/5 (1:1000), RUNX2 anti-bodies (1:1000), BMP-2 anti-bodies (1:1000), and COL1 anti-bodies (1:1000) were purchased from Cell Signaling Technology (Shanghai, China). Finally, horseradish peroxide (HRP) secondary antibody was added to the sample and incubated for 1 h. Analysis was then performed with the Odyssey infrared imaging system (LI-COR Biosciences, Lincoln, NE).

### Construction of animal model

All experimental procedures were approved by the Institutional Animal Care and Use Committee (IACUC) of the Shanghai Ninth People's Hospital, Shanghai Jiao Tong University School of Medicine. All mice were provided by the Research Council of the Animal Center of the Shanghai Ninth People's Hospital (China). All mice were kept at room temperature of 20–26 °C, with a relative humidity of 70% and light intensity ≥ 200 lx (12 h light/dark) and provided with sufficient food and water. MDA-MB-231 cells (5 × 10^7^ metastatic breast cancer cells) were subcutaneously injected at the underarm of the mice. Mice with a tumour size of 6 mm in diameter were randomly divided into 4 groups (n = 4): control, CS, CePO4/CS, CePO4/CS/GO groups. An incision was performed on the edge of the tumour, and the scaffold was installed. All mice were exposed to NIR radiation (0.55 W/cm) for 10 min. The tumour tissue temperature was monitored by a LIRTM A320 camera. All mice were exposed to NIR radiation once a day for 14 days. After 14 days, the mice were given full anaesthesia, and the immunofluorescence intensity of the metastatic breast cancer cells was measured with IVIS scanning (PerkinElmer, USA). At the end of the process, all mice were euthanized, and the tumour tissues were removed, followed by the measurement of the volume. After the quantitative measurement, the tumour tissues were fixed in 4% PFA, embedded in paraffin, and sliced, and the sliced tissues from each group were subjected to caspase-3 immunofluorescence staining.

Another animal model was initiated using Sprague–Dawley rats with defects in the skull. Female Sprague–Dawley rats of 200–250 g were selected as the model animal. An animal model with bilateral bone defects in the skull was used to measure the polarization of macrophages and the regeneration of bone. The diameter and height of the bone defects were 5 and 2 mm, respectively. CS, CePO_4_/CS, and CePO3/CS/GO scaffolds (n-5) were filled into the defects, and the scalp was sutured. The new bone formation and osteogenesis were examined by multicolour immunofluorescence. Intraperitoneal injections of the immunofluorescence markers alizarin red (30 mg/kg, Sigma-Aldrich) and calcein (30 mg/kg, Sigma-Aldrich) were given 3 and 21 days before sacrificing the rats, respectively. All rats were euthanized after 12 weeks, and the skulls with the scaffolds in the defects were dissociated. The skulls were stored in 4% phosphate formaldehyde solution for 7 days and subjected to micro-CT (mCT-80, Scanco Medical AG, Switzerland) examination. The parameters were as follows: a voltage of 90 kV, a current of 88 μA, and a pixel size of 28 μm. The 3D model was reconstructed after the scanning. The bone mineralization density (BMD) and new bone volume/tissue volume (BV/TV) were analysed by software. The undecalcified samples were fixed by bone cement, and the skulls were sectioned along the sagittal plane using a microtome (Leica, Hamburg, Germany). The immunofluorescence was measured by scanning confocal microscopy (Leica, Heidelberg, Germany; Alizarin red: 543/580–670 nm, Calcein: 488/500–550 nm). The bone mineralization rate was measured by a PC-based analysis system. The skulls were stored in 10% ethylene diamine tetraacetic acid (EDTA) for 30 days after the removal of the soft tissues. Finally, we dewaxed the sample and placed it in: xylene for 5 min, xylene for 5 min, xylene for 5 min, 100% ethanol for 2 min, 100% ethanol for 2 min, 100% ethanol for 2 min, and 90% ethanol for 2 min. 80% ethanol for 2 min; deionized water for 2 min. Put in the hematoxylin dye solution for 5 min. Eosin stained for 5 min. Subsequently, it was taken out and placed in 80% ethanol for 2 min; 90% ethanol for 2 min; 100% ethanol for 2 min; 100% ethanol for 2 min; xylene for 5 min; and 100% ethanol for 2 min. Hematoxylin–eosin (HE) staining and Masson staining were performed for a morphology analysis. The above samples were washed with TBST, and the dewaxed sections were taken out and placed in a blocking solution for 1 h on a shaker. After the end of the time, the blocked sections were washed 3 times with TBST for 10 min each time. Then carry out a primary antibody reaction. The antibody of the target protein was prepared in advance, and the primary antibody (iNOS, 1:200; CD206, 1:200) was diluted with the primary antibody dilution according to the instructions, and dropped on the surface of the slice. The primary antibody was applied and allowed to stand overnight at 4 °C. After the end of the primary antibody, the primary antibody was collected and the membrane was washed with TBST solution for 10 min each time. Finally, the secondary antibody was incubated, protected from light, and allowed to stand at room temperature for 2 h. The polarization index of the sample was detected by fluorescence microscopy.

### Data analysis

SPSS 13.0 software (Statistical Package for the Social Science, USA) was used to analyze the data. It presented as the Means ± SD. One-way analysis of variance was used to determine statistically significant differences. *P* ≤ 0.05 as statistically significant.

## Results

### Morphology and structure of CePO_4_ nanorods

**Fig. 1:**
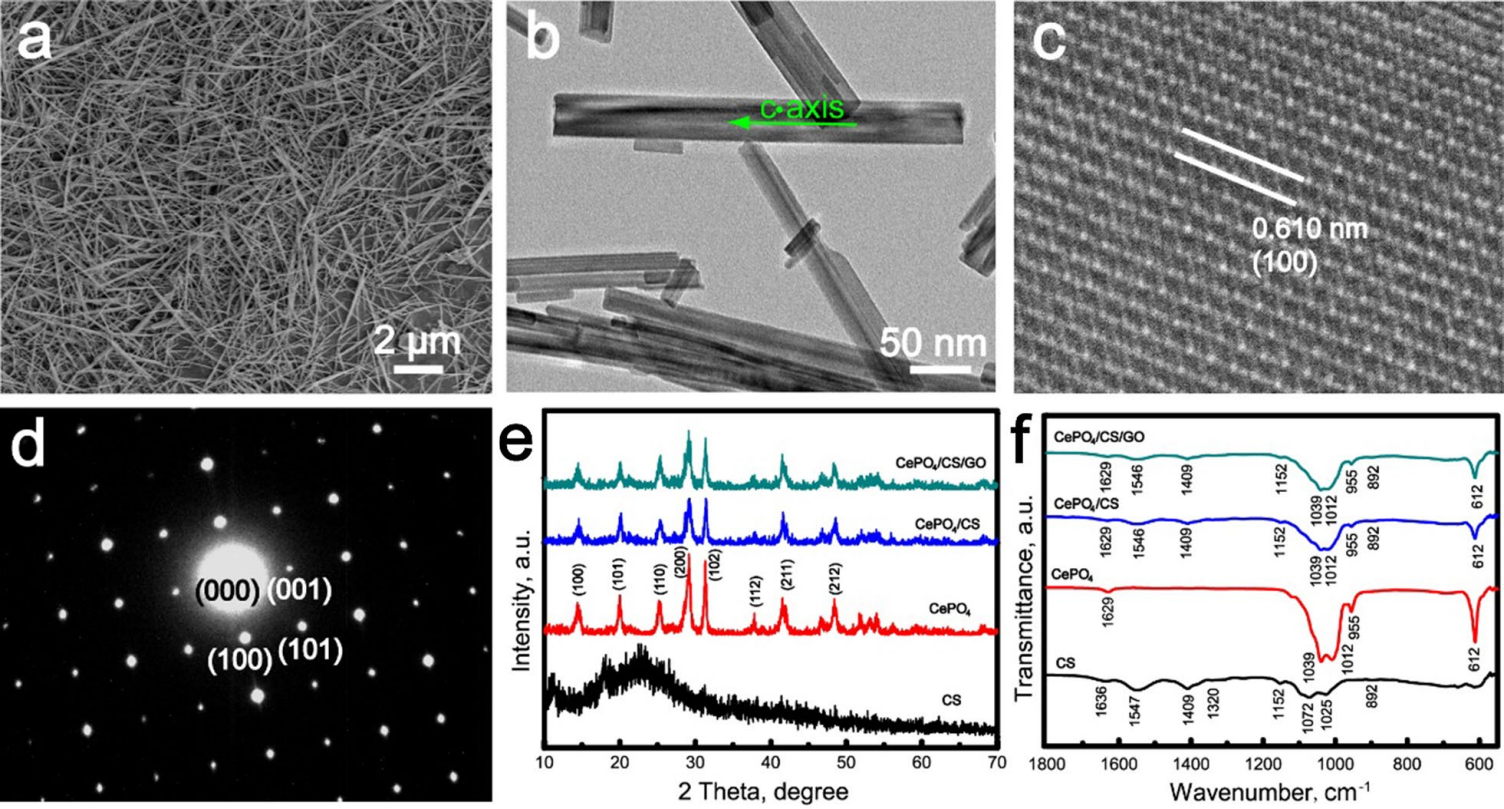
**a** SEM image, **b** TEM image, **c** HRTEM image and **d** SAED pattern of CePO_4_ nanorods. **e** XRD patterns and **f** FTIR spectra of CePO_4_ nanorods, CS scaffolds, CePO_4_/CS scaffolds and CePO_4_/CS/GO scaffolds

The CePO_4_ nanorods were prepared according to the hydrothermal method, as shown in Fig. 1. During the hydrothermal reaction, the CePO_4_ crystals grew along the *c*-axis orientation, forming one-dimensional rod shapes. The CePO_4_ nanorods had diameters of 5 µm and lengths of 50 nm, as confirmed by the SEM and TEM images (Fig. 1a, b). The crystal structure of CePO_4_ nanorods was investigated further by the high-resolution TEM images (Fig. 1c). The lattice fringes of the CePO_4_ nanorods demonstrated the monocrystalline structure, and the *d*-space of 0.610 nm was ascribed to (100) crystal plane. The SAED pattern of CePO_4_ nanocrystals revealed that the diffraction spots corresponded to [010] zone axis (Fig. 1d).

The phase structure of CePO_4_ nanorods was investigated by XRD pattern (Fig. 1e). All the diffraction peaks were much matched with the hexagonal CePO_4_ crystals with space group of P622 (180), which were indexed to PDF card NO. 34-1380. The cell parameters of CePO_4_ nanorods were calculated by using MDI JADE5.0 software, as followed: *a* = *b* = 0.7067 nm, *c* = 0.6439 nm, *α* = *β* = 90º, and *γ* = 120º. The characteristic peaks of CePO_4_ nanorods showed strong peak intensity, demonstrating their high crystallinity. Moreover, the functional groups in CePO_4_ nanorods were revealed by FTIR spectrum (Fig. 1f). The band at 1619 cm^−1^ was ascribed to adsorbed water on the surfaces of CePO_4_ nanorods. The bands due to the asymmetric stretching vibration of PO_4_^3−^ groups located at 1039 and 1012 cm^−1^. The P–O bending vibration and antisymmetric deformation vibration band located at 955 cm^−1^ and 620 cm^−1^, respectively.

### Characterization of CePO_4_/CS/GO scaffolds

**Fig. 2:**
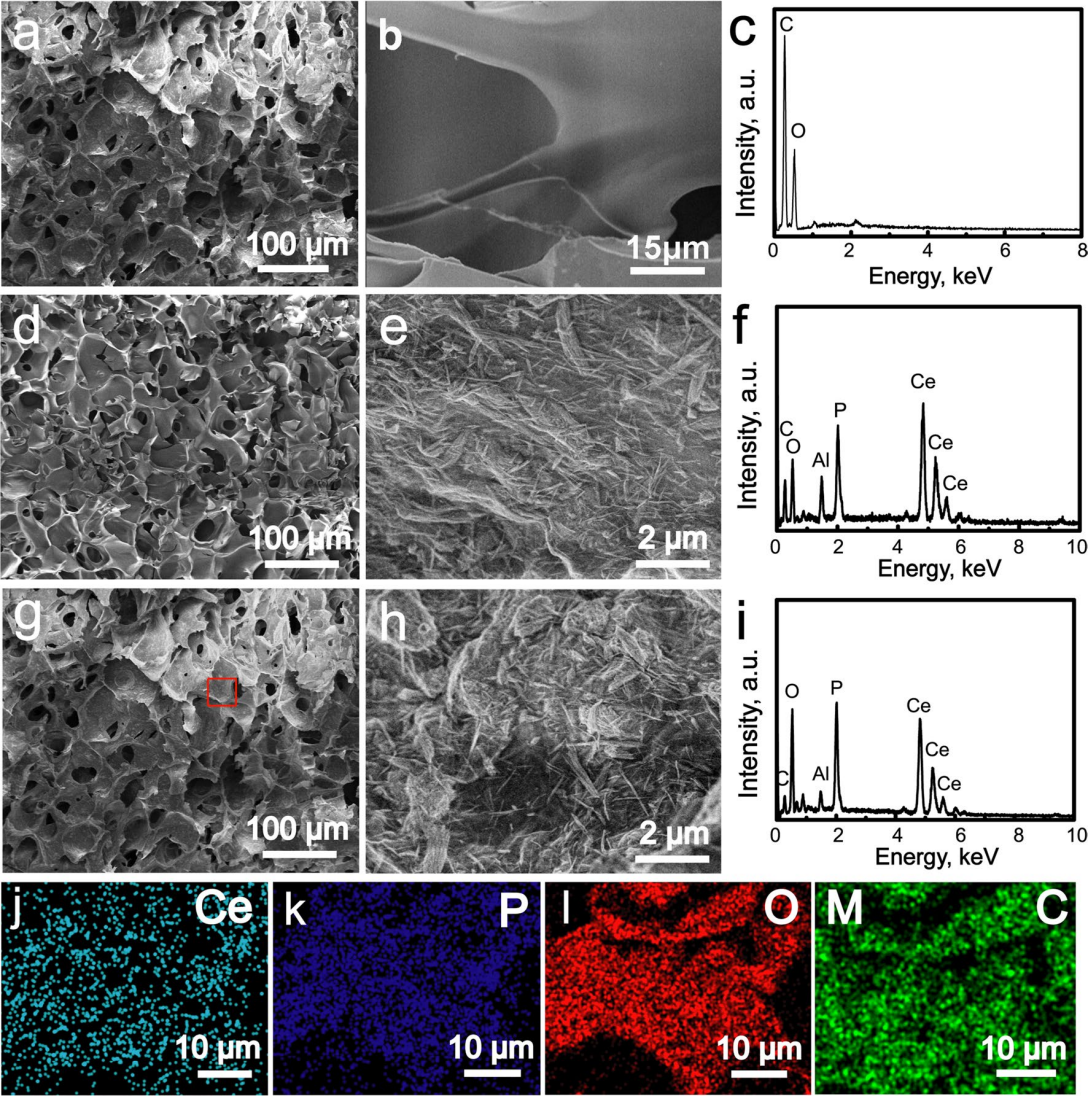
**a**, **b** SEM images and **c** EDS pattern of CS scaffolds; **d**, **e** SEM images and **f** EDS pattern of CePO_4_/CS scaffolds; **g**, **h** SEM images, **i** EDS pattern, **j**–**m** Ce, P, O and C element distribution images of CePO_4_/CS/GO scaffolds which corresponded to the red block in image (**g**)

The freeze-drying technology was used to construct three-dimensional porous scaffolds including CS, CePO_4_/CS and CePO_4_/CS/GO scaffolds. All CS, CePO_4_/CS and CePO_4_/CS/GO scaffolds had the 3D macropores with sizes of 80 µm (Fig. 2a, d and g), which were created due to ice crystal sublimation during the freeze-drying procedure. These macroporous structures could provide enough spaces for cell migration, nutrient transfer and bone tissue ingrowth. Interestingly, these macropores existed among the plate-like films. For the pure CS scaffolds, the films presented smooth surfaces (Fig. 2b). For the CePO_4_/CS and CePO_4_/CS/GO scaffolds, many CePO_4_ nanorods were uniformly dispersed on the films (Fig. 2e and h). The GO nanosheets were not obviously detected in the CePO_4_/CS/GO scaffolds because of small particle sizes and low percentages (Fig. 2 h). The chemical elements in the CS, CePO_4_/CS and CePO_4_/CS/GO scaffolds were detected by EDS patterns (Fig. 2c, f and i). The Ce and P were originated from the CePO_4_ nanorods, C was originated from CS or/and GO (Fig. 2c, f and i). Notably, the Ce, P and C element distribution maps further demonstrated that the CePO_4_ nanorods and GO nanosheets were uniformly distributed throughout the CePO_4_/CS/GO scaffolds (Fig. 2j–m).

**Fig. 3:**
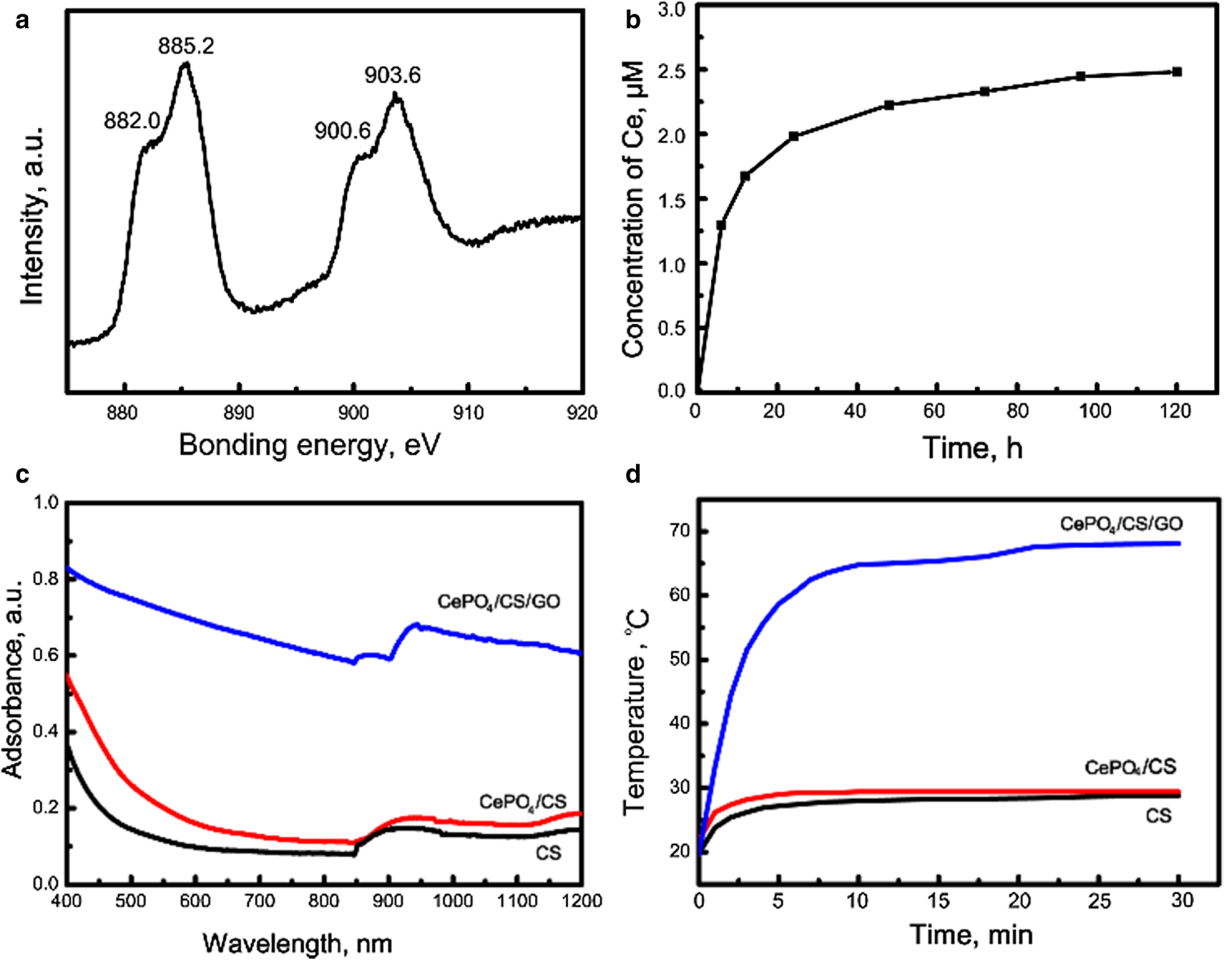
**a** Ce3d XPS pattern of CePO_4_ nanorods in CePO_4_/CS/GO scaffolds; **b** Ce^3+^ ion release trend from CePO_4_/CS/GO scaffolds; **c** UV–vis spectra of CS, CePO_4_/CS and CePO_4_/CS/GO scaffolds; and **d** temperature change trends with the irradiation time of NIR light for the CS, CePO_4_/CS and CePO_4_/CS/GO scaffolds

The XRD patterns of the CePO_4_/CS and CePO_4_/CS/GO scaffolds was much similar to the pure CePO_4_ nanorods (Fig. 1e). Notably, only CePO_4_ characteristic peaks were detected in the XRD patterns of CePO_4_/CS/GO scaffolds, which was ascribed to the following two reasons: (i) CS showed amorphous characteristic, as confirmed by the XRD pattern of CS scaffolds (Fig. 1e); (ii) the percentage of the GO nanosheets in the scaffolds was only approximately 2.9%. The functional groups of the CePO_4_/CS and CePO_4_/CS/GO scaffolds were demonstrated by FTIR spectra (Fig. 1f). The characteristic bands of CS were detected in the FTIR spectra of the CS, CePO_4_/CS and CePO_4_/CS/GO scaffolds, including C = N vibration (1636 cm^−1^), C-N vibration (1409/1320 cm^−1^), bridge oxygen stretching vibration (1152 cm^−1^), N–H deformation vibration (1547 cm^−1^), C-O stretching vibration (1025/1072 cm^−1^), N–H wagging vibration (892 cm^−1^). The characteristic bands of CePO_4_ nanorods were detected in the FTIR spectra of the CePO_4_/CS and CePO_4_/CS/GO scaffolds, too (Fig. 1f). The bands due to GO were not observed in the FTIR spectrum of the CePO_4_/CS/GO scaffolds due to the low percentages, which was in good agreement with the XRD results (Fig. 1e, f). Figure 3a showed the Ce3d XPS pattern of CePO_4_ nanorods in the CePO_4_/CS/GO scaffolds. The peaks at 882.1 and 885.8 eV corresponded to the Ce3d_5/2_, and those at 900.5 and 903.9 eV corresponded to Ce3d_3/2_. All the above peaks were assigned to Ce^3+^ ions, suggesting that the Ce ions in the CePO_4_ nanorods existed as trivalent states without Ce^4+^.

In order to assay the release performances of CePO_4_/CS/GO scaffolds in vitro, the samples were immersed in ultrapure water. With increasing the soaking time, the Ce^3+^ ions were continuously released from the scaffolds (Fig. 3b). Notably, the Ce^3+^ ions showed quick release performance within 48 h, and then gradually arrived at the dissolution-reprecipitation balance. After the release for 120 h, the concentrations of Ce^3+^ ions reached 2.48 μM. The high crystallinity of the CePO_4_ nanorods caused the low degradability, resulting in the slow release rates of Ce^3+^ ions (Figs. 1e and 3b).

The mechanical property of CePO_4_/CS/GO scaffolds was characterized by microcomputer control electronic universal testing machine, as shown in Additional file 1: Figure S1. Since CS scaffolds possessed a ductile feature, three-dimensional porous structure was easy to cause damage under load-bearing conditions [39]. The combination of CePO_4_ nanorods, CS and GO formed the organic/inorganic hybrid scaffolds, leading to a good mechanical property. As the external forces were exerted on the CePO_4_/CS/GO scaffolds, the macropore damage and material deformation successively took place (Additional file 1: Figure S1). When the compressive strengths of the CePO_4_/CS/GO scaffolds arrived at approximately 0.21 MPa, the macroporous structure was completely destroyed.

As we know, GO possesses excellent light‐absorbing properties and photothermal conversion efficiency. The incorporation of GO nanosheets provided the CePO_4_/CS/GO scaffolds with better light adsorption properties in the region of 400–1200 nm than the CS and CePO_4_/CS scaffolds (Fig. 3c). With increasing the irradiation time of NIR light, the temperatures in the CS and CePO_4_/CS scaffolds rose slightly (Fig. 3d). Interestingly, the temperatures around the CePO_4_/CS/GO scaffolds rose rapidly, and arrived at 64.8 ºC after only 10 min, which was high enough to kill tumor cells.

### Effects of different scaffold materials against bone metastasis from breast cancer under nir exposure

**Fig. 4.**
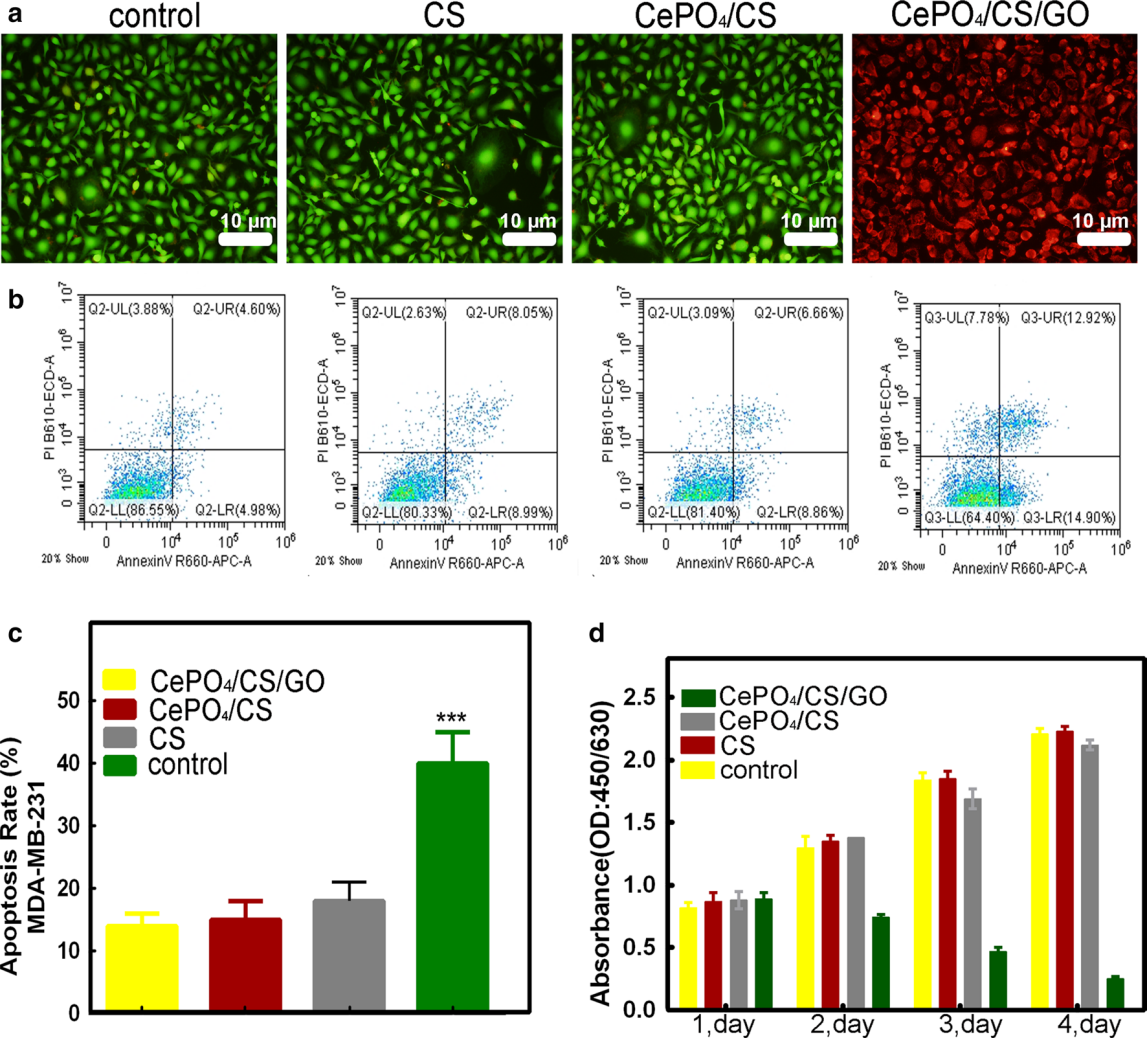
**a** live-dead cell staining, **b**, **c** flow cytometry of MDA-MB-231 cells for blank, CS, CePO_4_/CS and CePO_4_/CS/GO groups under NIR laser irradiation for 10 min every day. **d** CCK-8 analyses

Figure 4a, b shows the result by live-dead cell staining and flow cytometry cell counting. It was found that the MDA-MB-231 cells in the control, CS and CePO_4_/CS control groups were live cells, while those in the CePO_4_/CS/GO group were dead cells (green represents live cells and red represents dead cells). The CePO_4_/CS/GO scaffolds significantly increased the local temperature under NIR light compared to the groups without GO nanoparticles. Figure 4c illustrates the results of the statistical analysis of the flow cytometry. The degree of apoptosis resulting from the NIR exposure was different in each group, with the highest number of dead cells in the CePO_4_/CS/GO group. Figure 4d shows the proliferation of MDA-MB-231 cells cultured with different scaffolds while under NIR exposure. From day 1 to day 4, the survival of the control, CS and CePO_4_/CS groups increased with the culturing time, but the survival of the CePO_4_/CS/GO group decreased. This result suggested that the proliferation of MDA-MB-231 cells in the CePO_4_/CS/GO group was inhibited under NIR exposure.

**Fig. 5:**
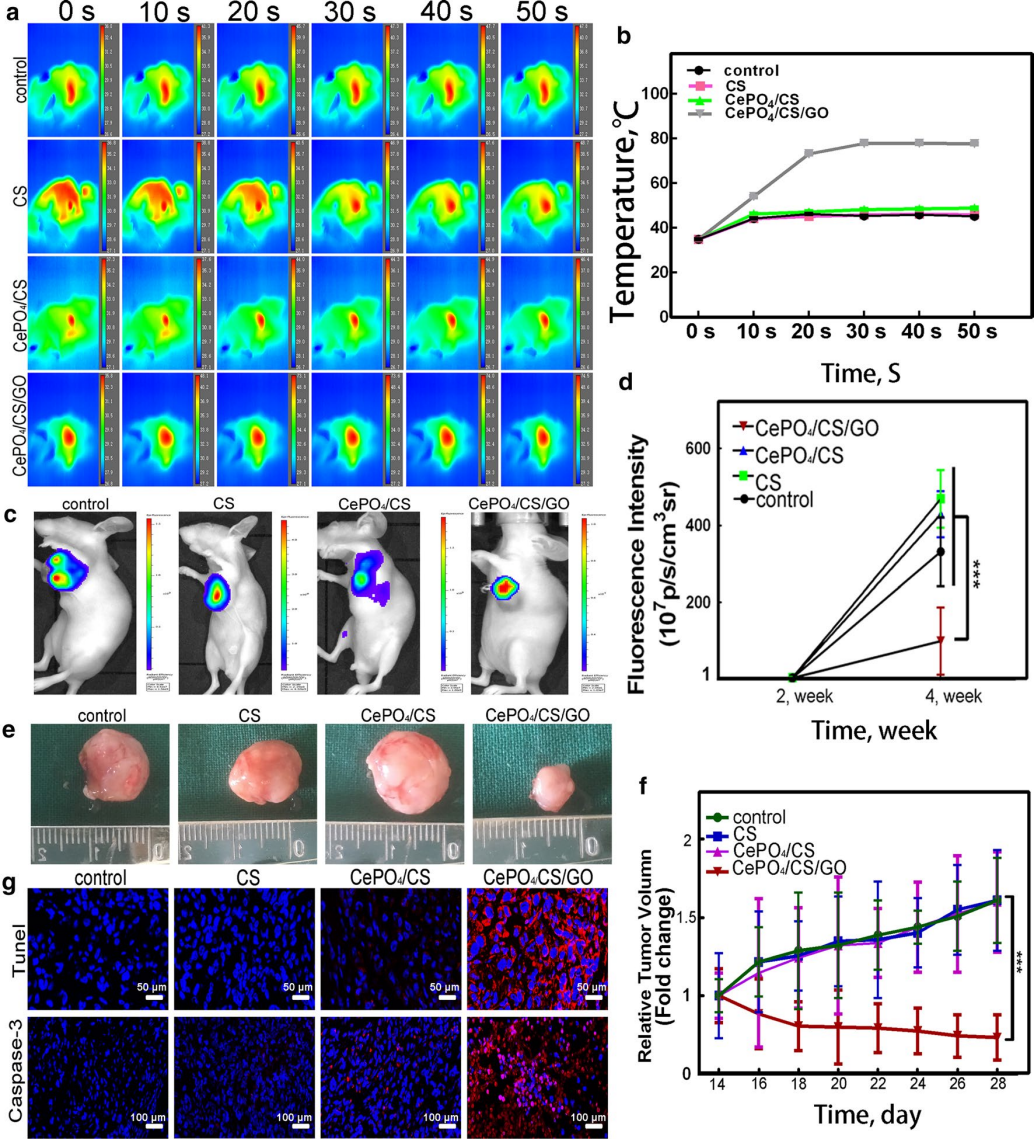
**a**, **b** The temperature changes after exposure to NIR radiation. **c**, **d** Fluorescence detection on nude mice after NIR laser irradiation by IVIS Lumina K Series III and fluorescence intensity of the CePO_4_/CS/GO group was significantly lower than the blank, CS and CePO_4_/CS groups. **e**, **f** Optical picture of tumors in nude mice, and quantitative analysis of tumor volume. **g** Histomorphological observation of tumors. Tunel represented apoptosis (blue: nucleus, red: apoptosis), and Caspase-3 represented the most important terminal cleavage enzyme in the process of apoptosis

In vivo, Scaffolds with different materials were then implanted into the mice. All scaffolds were exposed to NIR light within 30 s. The temperature of the implanted scaffold was measured by thermal imaging (Fig. 5a). As shown in Fig. 5b, the temperature in the CePO_4_/CS/GO group was higher than those in the other groups: the temperature reached as high as 52 degrees, while the highest among the other groups was only 41 degrees. After the second week, IVIS Lumina K Series III was employed for florescence measurements on the mice. The florescence intensity was measured again after 2 weeks of NIR exposure (Fig. 5c). Compared with the control, CS and CePO_4_/CS groups, the red fluorescence intensity in the CePO_4_/CS/GO group began to decrease with prolonging the therapy time (Fig. 5d). This result indicated that CePO_4_/CS/GO could significantly inhibit tumour cell proliferation. After the measurements, the mice were euthanized, followed by the removal of the tumours and the measurement of the tumour sizes. As shown in Fig. 5e, f the tumour size of the CePO_4_/CS/GO group was significantly smaller than those of the other three groups. Figure 5 g shows the TUNEL staining and caspase-3 staining of the tumours, which reveal the degree of the tumour cell apoptosis. Apoptosis was observed in the CePO_4_/CS/GO group but not in the other group.

### Effects of different scaffold materials on the degree of polarization of RAW264.7 cells

**Fig. 6:**
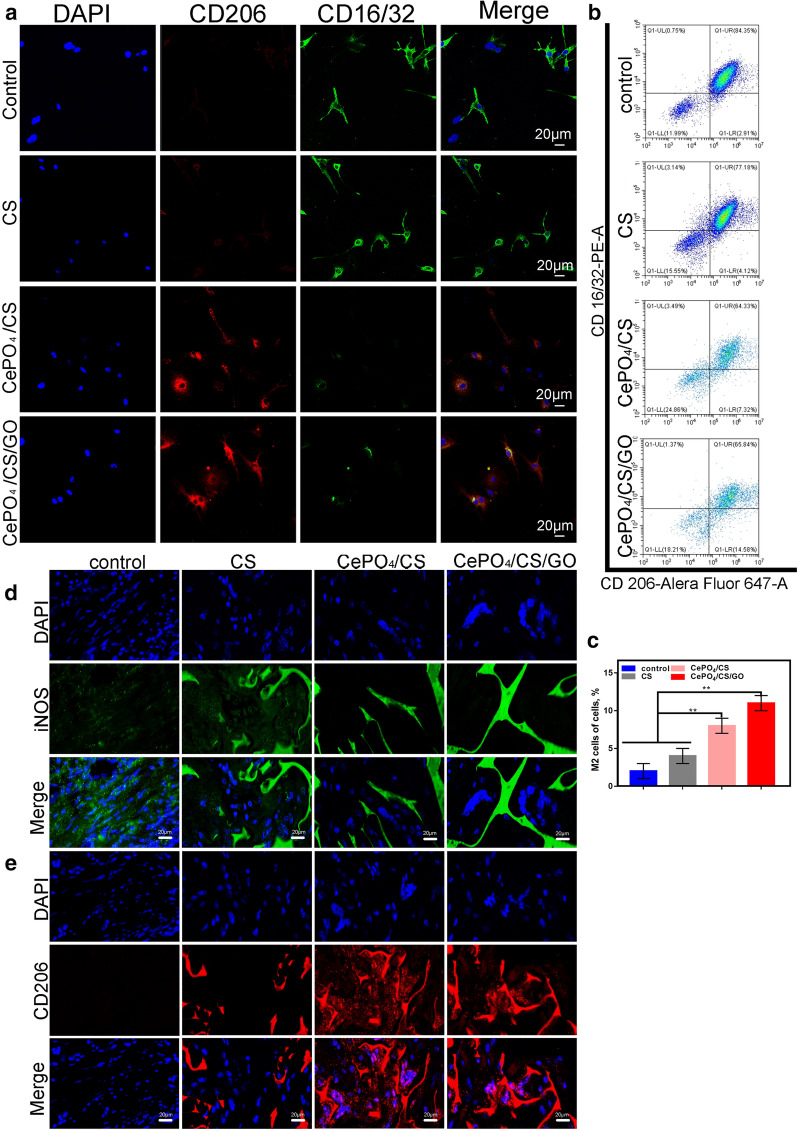
**a** Immunofluorescence images of RAW264.7 macrophages co-cultured with different scaffolds. The nucleus, M2 macrophage and M1 macrophage staining for DAPI, CD206 and CD16/32, respectively. **b**, **c** Flow cytometry. **d**, **e** In vivo, macrophages expressed iNOS proteins in the control and CS groups, while macrophages expressed CD206 proteins in the CePO_4_/CS, and CePO_4_/CS/GO groups

The RAW cells were cultured with control, CS, CePO4/CS, and CePO4/CS/GO scaffolds. After 24 h of culture, RAW cells were added with the primary antibodies (CD206 represents M2 macrophages and CD16/32 represents M1 macrophages) and incubated overnight. The color-labeling secondary antibodies were added and incubated for 1 h, and the samples were then observed with a confocal microscope. The immunofluorescence intensities of the CePO4/CS and CePO4/CS/GO were observed (Fig. 6a), and the florescence intensity of CD206 was significantly higher than those of the other two groups, which suggested that the cells of these groups polarized into M2 macrophages. The results of the flow cytometry are also provided (Fig. 6b, c), which shows the effects of different scaffold materials on the polarization of macrophages. It was found that the macrophages did not differentiate into M2 macrophages in the control and CS groups, but they did in the presence of Ce. Figure 6d, e illustrates the results of different scaffolds on the polarization of macrophages. In vivo experiments indicate that the control and CS group do not promote macrophage polarization in the M2 direction. However, the scaffold material of the CePO_4_/CS and CePO_4_/CS/GO groups can promote the M2 direction polarization of macrophages. We know that M2 macrophages can promote the formation of blood vessels [34, 36]. and promote the mineralization of osteoblasts [35, 37].

### Study on the toxicity and osteo-inductivity of scaffold materials to MC3T3-E1 cells

**Fig. 7:**
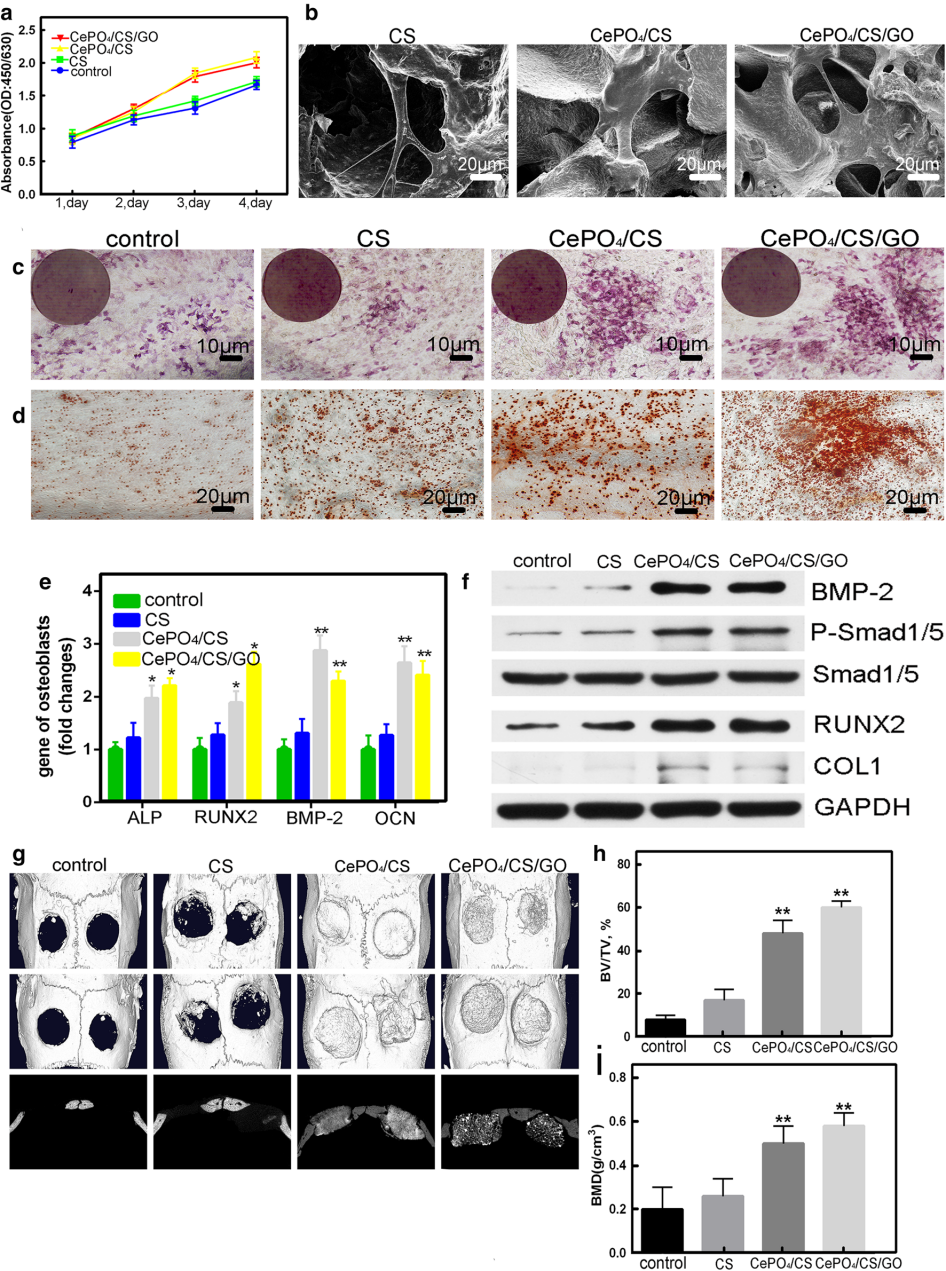
**a** CCK-8 assays of MC3T3-E1 cells cultured in the extracts of different scaffold materials for different days. **b** SEM images of hBMSCs co-cultured on CS, CePO_4_/CS and CePO_4_/CS/GO scaffolds. **c**, **d** ALP staining and alizarin red staining images of MC3T3-E1 cells cultured in the extracts of CS, CePO_4_/CS and CePO_4_/CS/GO scaffolds for 7 days. **e** RT-PCR analysis for ALP, RUNX2, BMP-2 and OCN expression of MC3T3-E1. **f** Western blot analysis for BMP-2, P-Smad1/5, Smad1/5, RUNX2 and COL1 of MC3T3-E1. **g** Micro-CT images of skulls of the control, CS, CePO_4_/CS, and CePO_4_/CS/GO groups 3 months after the surgery. **h** Bone volume/tissue volume (BV/TV). **i** Quantitative analysis of the morphology of the bone mineralization density (BMD), n = 3, *P < 0.05

The freeze-drying technology was employed to fabricate the CePO_4_/CS/GO porous scaffolds, in which CePO_4_ nanorods and GO nanosheets were dispersed through the whole scaffolds (Fig. 1). Rare earth elements (REEs), for example Ce, could accumulate in human bones mainly by substituting parts of Ca^2+^ ions in apatite. The biological responses of the REEs represented the hermetic concentration–response relationship, namely, the low concentrations showed positive stimulation and the high concentrations showed inhibition effects against normal cells. Fortunately, the CePO_4_/CS/GO scaffolds had the controlled release performances of Ce^3+^ ions because of the appropriate biodegradability, and the Ce^3+^ concentrations were kept at only 2.48 μM even after 120 h. The low concentrations of Ce^3+^ ions did not do harm to hBMSCs and MC3T3-E1 cells, and even contributed to cell proliferation (Fig. 7a). Moreover, the 3D microporous in the scaffolds facilitated the cell migration, and the degree of the hBMSCs’ adhesion on the surface of the CS, CePO_4_/CS, and CePO_4_/CS/GO scaffolds and their morphological characteristics were observed by SEM (Fig. 7b). In vitro assays demonstrated that the CePO_4_/CS/GO porous scaffolds had excellent biocompatibility.

The effects of the CS, CePO_4_/CS, and CePO_4_/CS/GO scaffolds on osteo-inductivity were studied via ALP staining, alizarin red staining, PCR, and western blot. An ALP staining image of the MC3T3-E1 cells of the control group is provided in Fig. 7c. The cells of this group were cultured with CS, CePO_4_/CS, and CePO_4_/CS/GO for 7 days and extracted. The number of ALP-stained cells of the CePO_4_/CS and CePO_4_/CS/GO group was significantly higher than those of the control and CS groups. Figure 7d illustrates the calcium deposition of the groups cultured with the control, CS, CePO_4_/CS, and CePO_4_/CS/GO scaffolds. Compared to the CS and control groups, the calcium deposition of the CePO_4_/CS and CePO_4_/CS/GO groups was significantly increased. The osteoblast activity of the CS, CePO_4_/CS, and CePO_4_/CS/GO groups was analysed by RT-PCR based on the expression levels of the ALP, BMP-2, OCN, and RUNX2 genes in the early osteoblasts (Fig. 7e). The expression levels of the ALP, BMP-2, OCN, and RUNX2 genes were the highest in the CePO_4_/CS, and CePO_4_/CS/GO group. Further, the western blot results of the osteogenesis-associated proteins are provided in Fig. 7f. The expression levels of the BMP-2, P-Smad1/5, Smad1/5, RUNX2 and COL1 proteins were higher in the CePO_4_/CS and CePO_4_/CS/GO groups compared to the other groups. In vivo, it was seen in the micro-CT images that the volume of newly generated bone tissue was higher in the CePO_4_/CS and CePO_4_/CS/GO groups than in the control group. Compared to CS, CePO_4_/CS and CePO_4_/CS/GO could effectively promote the formation of new bone (Fig. 7 g). A quantitative analysis of the morphology of the CePO_4_/CS (48.15 ± 4.21%) and CePO_4_/CS/GO (58.67 ± 3.32%) groups showed that the bone volume/tissue volume (BV/TV) ratios were higher than those of the control group (9.81 ± 3.26%) and CS group (21.21 ± 2.95%) (Fig. 7 h). Compared to the control and CS groups, the bone mineral densities of the CePO_4_/CS and CePO_4_/CS/GO groups were higher (Fig. 7i).

**Fig. 8:**
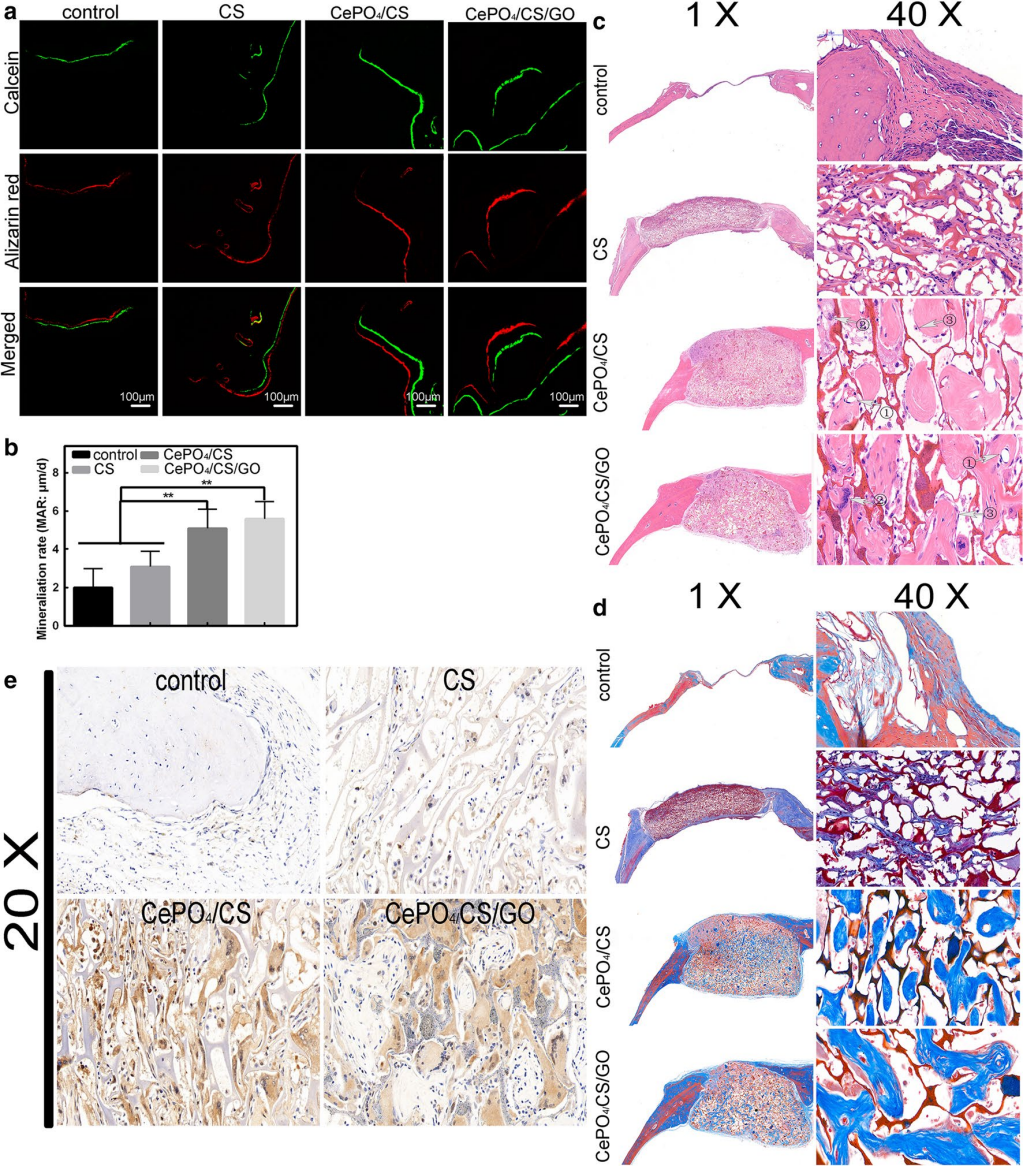
**a** Line 1 (green) represents the calcein 3 weeks prior to sacrificing the mice. Line 2 (red) represents the alizarin red 3 days prior to sacrificing the mice. Line 3 represents the combination of the two florescence lines. **b** Quantitative analysis of the two florescence lines. **c**, **d** illustrates the results of HE staining and Masson staining for the investigation of the bone and collagen components of the control, CS, CePO_4_/CS, and CePO_4_/CS/GO groups. Figure 8c ① a large number of osteoblasts are adhered to the surface of the bone matrix. Figure 8c ② a large number of macrophages are aggregated near the material. Figure 8c ③ new blood vessels formed by vascular endothelial cells. Figure 8e immunohistochemical analysis of VEGF of CePO_4_/CS and CePO_4_/CS/GO groups

To investigate the mineralization of osteoblasts, the florescence labelling agents alizarin red and calcein were injected at different times prior to sacrificing the mice. The first line represents data from calcein, which suggests that bone mineralization occurred 21 days prior to sacrificing the mice. The second line represents data from alizarin red, which suggests that bone mineralization occurred 3 days prior to sacrificing the mice. The average distance between the two lines represent the newly formed bone tissue (Fig. 8a). The bone mineralization rate was analysed by a PC-based analysis system, and the calculated ratios for the CePO_4_/CS and CePO_4_/CS/GO groups, 5.38 ± 0.68 μm/d and 6.54 ± 0.52 μm/d, respectively, were significantly higher than those of the CS (2.34 ± 0.32 μm/d) and control groups (1.98 ± 0.48 μm/d) (Fig. 8b). Figure 8c, d illustrate the results of the HE staining and Masson staining for the investigation of the bone and collagen components of the four groups. An immunohistochemical analysis of VEGF was performed. This property was confirmed by immunohistochemical analysis of VEGF in tissues (Fig. 8e). It was found that the amount of VEGF proteins was significantly increased in the CePO_4_/CS and CePO_4_/CS/GO groups compared with the other two groups.

## Discussion

Metastatic cancer cells can grow on the surface of bone tissue and can damage otherwise normal bone tissue. Surgical removal of bone tissue followed by chemotherapy is the primary treatment, once an osteolytic lesion is found. However, surgical removal of bone tissue can cause bone defects, while radiotherapy/chemotherapy leads to a reduction of physical function. These side effects can create difficulties in the fusion of bone and implant materials or even the occurrence of a non-union. Therefore, it is necessary to create a biomaterial that can solve these issues. This material should have the ability to kill residual tumors, promote fusion between osteoblasts and materials, and promote the growth of osteoblasts in materials. For combined treatment of tumors, Wang, SG. Scholars reported on thermotherapy technology before [40, 41].

Graphene oxide (GO) with excellent light‐absorbing property and photothermal conversion efficiency has been widely used for PTT application [42, 43]. The main characteristic of the material we are focusing on is the limination of residual tumor effects. The scaffolds should provide thermal effects and GO nanosheets have an excellent NIR absorption and photothermal conversion efficiency [44, 45]. The CePO_4_/CS/GO scaffold temperatures increased to 51.4 ℃ after 5 min of NIR laser irradiation. This phenomenon indicates that the GO in the scaffold converts NIR light into heat energy, and then conducts heat energy to the scaffolds to generate thermal energy as a whole, which effectively kills the cancer cells. In contrast, under the NIR light irradiation, the temperature of scaffolds without GO nanoparticles has no significant increase. Compared with the control, CS and CePO_4_/CS groups, the CePO_4_/CS/GO scaffolds within the scope of NIR light irradiation significantly induced the tumor apoptosis of MDA-MB-231 cells.

Scaffold material was implanted into standard nude mice to further determine its photothermal therapy effect. MDA-MB-231 cells were also transfected with red fluorescent markers (red fluorescence intensity represents live MDA-MB-231 cells) followed by the injection of MDA-MD-231 cells (5 × 10^7^ cells) were injected at the underarm of the mice. The mice were randomly divided into 4 groups (n = 4) after the tumour reached an 8 mm diameter. According to our research, the thermal energy generated by the CePO_4_/CS/GO scaffold can activate caspase-3 protease, which plays an irreplaceable role in apoptosis. At the initiation of apoptosis, Caspase-3 separates the two zinc finger structures that bind to DNA in poly (ADP-ribose) polymerase (PARP) from the catalytic region at the carboxy terminus and does not function properly. Eventually, the activity of Ca^2+^/Mg^2+^-dependent endonuclease is increased, and DNA between nucleosomes is cleaved, causing apoptosis. On the contrary, the others did not have photothermal therapeutic effects for MDA-MB-231 cells. Therefore, the incorporation of GO nano-sheets makes the CePO_4_/CS/GO scaffold excellent in photothermia effect on tumors.

Designing of a different scaffold is also aimed at inhibiting the fibrous connection between scaffold and bone tissue. The formation of fibrous connective tissue is often facilitated by pro-inflammatory cytokines. Our investigation determined that macrophages can rapidly aggregate on the scaffold material instead of the formation of connective tissue promoted by pro-inflammatory cytokines. Several studies have shown that macrophages can be categorized into two types: pro-inflammatory macrophages and anti-inflammatory macrophages. Through microscopy and flow cytometry, it was found that the Ce^3+^ released from the CePO_4_/CS/GO scaffold could promote the polarization of macrophages. Macrophages can be categorized into M1 and M2 macrophages. M1 macrophages are pro-inflammatory macrophages that can release large amounts of pro-inflammatory cytokines, while M2 macrophages are anti-inflammatory macrophages that can release large amounts of anti-inflammatory cytokines. This study illustrated the as-released Ce^3+^ ions released from the scaffolds promotes the M2 polarization of macrophages. However, the macrophages in the control and CS groups exhibited M1 macrophages. Once the as-released Ce^3+^ ions released from the scaffolds, the M1 macrophages were inhibited, the fibrous tissue surrounding the prosthesis is inhibited, thus reducing the incidence of non-union. Moreover, M2 macrophages can promote rapid fusion between the bone tissue and scaffold material, which facilitates the growth of bone tissue in the scaffold [18, 38].

Finally, our concern is the promotion of bone repair by biomaterials. The freeze-drying technology was employed to fabricate the CePO_4_/CS/GO porous scaffolds, in which CePO_4_ nanorods and GO nanosheets were dispersed through the whole scaffolds (Fig. 1). Rare earth elements (REEs), for example Ce, could accumulate in human bones mainly by substituting parts of Ca^2+^ ions in apatite. From the above experimental results showed the CePO_4_/CS and CePO4/CS/GO scaffolds could promote the expression levels of the ALP, BMP-2, OCN, and RUNX2 genes and the osteogenesis-associated proteins. The expression levels of the BMP-2, P-Smad1/5, Smad1/5, RUNX2 and COL1 proteins were higher in the CePO_4_/CS and CePO_4_/CS/GO groups. It showed that the as-released Ce^3+^ ions activated BMP-2/Smad signaling pathway that facilitated bone tissue regeneration. In the CePO_4_/CS and CePO_4_/C/GO groups, a large number of osteoblasts was found (Fig. 8c, ①) to adhere to the surface of the bone matrix and were in a concentric circle orientation. A large number of macrophages aggregated around the material (Fig. 8c, ②), and new blood vessels were formed by vascular endothelial cells (Fig. 8c, ③). The centre was a blood vessel, which was similar to a bone-like structure (Harvard tube). Due to the larger pores, compared to the CS and control groups, there was more collagen filling the pores, as observed in the CePO_4_/CS and CePO_4_/CS/GO groups (Fig. 8d). Moreover, the larger pores seen in the CePO_4_/CS and CePO_4_/CS/GO groups were covered by the newly generated bone; the amount of newly generated bone was also higher than those of the other groups. M2 macrophages can facilitate the formation of blood vessels which promotes angiogenesis which provides oxygen and nutrient for osteogenesis. This property was confirmed by immunohistochemical analysis of VEGF in tissues. (Fig. 8e). It was found that the amount of VEGF proteins was significantly increased in the CePO_4_/CS and CePO_4_/CS/GO groups compared with the other two groups. One reason for angiogenesis in materials is that Ce^3+^ ions promotes the polarization of macrophages, which releases the VEGF factor. The VEGF factor is an essential factor in angiogenesis. Once blood vessels form in the material, the nutrient supply to the bone tissue in the scaffold is secured. These results confirmed that the scaffold material containing Ce^3+^ can promote the differentiation of osteoblasts.

## Conclusion

Graphene oxide (GO) nanoparticles, hydrated CePO_4_ nanorods and bioactive chitosan (CS) are combined to form a bioactive multifunctional CePO_4_/CS/GO scaffolds, which has the following characteristics such as photothermal therapy to kill tumors, macrophage polarization promotes blood vessel formation and induces bone formation. In the NIR laser irradiation, CePO_4_/CS/GO bracket activate local tumor cells caspase-3 protease, which cleavage of DNA between nucleosomes and to induce apoptosis. The hydrated CePO_4_ nanorods are used as novel bioactive components to enhance angiogenesis and osteogenic capacity. First, the Ce^3+^ ion M2 polarization of macrophages to promote the release of the release material of the stent, M2 macrophages and vascular endothelial growth factor (VEGF) and arginase-1 (Arg-1), which facilitates Angiogenesis and provides osteogenesis oxygen and nutrients. Second, the as-released Ce^3+^ ions also activated BMP-2/Smad signaling pathway that facilitated bone tissue regeneration. Hence, the multifunctional CePO_4_/CS/GO scaffolds kill residual bone tumor cells after photothermal therapy and subsequent bone defect healing, which may become a promising platform for therapy of breast cancer bone metastases.

## Supplementary Information

**Additional file 1:** **Figure S1.** Compression properties of CePO_4_/CS/GO scaffolds (n=3).

## Data Availability

The data that support the findings of this study are available from the corresponding author upon reasonable request.

## Abbreviations

GO: Graphene oxide
CS: Chitosan
VEGF: Vascular endothelial growth factor
Arg-1: Arginase-1
PTT: Photothermal therapy
NIR: Near-infrared
REEs: Rare earth elements
Ce: Cerium
MSCs: Mesenchymal stem cells
SAED: Selected area electron diffraction
SEM: Scanning electron microscopy
EDS: Energy-dispersive spectrometry
XRD: X-ray diffractometer
FTIR: Fourier transform infrared spectroscopy
XPS: X-ray photoelectron spectroscopy
ICP/OES: Inductively coupled plasma/optical emission spectrometry
hBMSCs: Bone marrow mesenchymal stem cells
α-MEM: α-Minimum essential medium
FBS: Foetal bovine serum
CCK-8: Cell Counting Kit-8
PBS: Phosphate-buffered saline
ALP: Alkaline phosphatase
PFA: Paraformaldehyde
HRP: Horseradish peroxide
IACUC: Institutional Animal Care and Use Committee
BMD: Bone mineralization density
BV/TV: New bone volume/tissue volume
EDTA: 10% Ethylene diamine tetraacetic acid
