# Correction to: Graphene-modified CePO_4_ nanorods effectively treat breast cancer-induced bone metastases and regulate macrophage polarization to improve osteo-inductive ability

**DOI:** 10.1186/s12951-021-00829-0

**Published:** 2021-03-30

**Authors:** Yu-Wei Ge, Xiao-Liang Liu, De-gang Yu, Zhen-An Zhu, Qin-Fei Ke, Yuan-Qing Mao, Ya-Ping Guo, Jing-Wei Zhang

**Affiliations:** 1 Shanghai Key Laboratory of Orthopedic Implants, Department of Orthopedic Surgery, Shanghai Ninth People’s Hospital, Shanghai JiaoTong University School of Medicine, Shanghai, 200011 China; 2 The Education Ministry Key Lab of Resource Chemistry and Shanghai Key Laboratory of Rare Earth Functional Materials, Shanghai Normal University, Shanghai, 200234 China

## Correction to: J Nanobiotechnol (2021) 19:11 https://doi.org/10.1186/s12951-020-00753-9

Following publication of this article [1] the authors identified mistakes in Fig. 2a, d, g (and its caption) and Fig. 5g. Errors in the representative SEM images of CS scaffolds, CePO_4_/CS scaffolds and CePO_4_/CS/GO scaffolds were found, which were possibly made during image collection. The correction of these figures does not affect the results and conclusion of the article and all authors agree to these corrections.

The incorrect and correct Figs. 2 and 5 are published in this Correction article. The original article has been updated.

**Figure 2 before correction** (Fig. 2a, d, g contained an error caused by the disordered sequence of pictures before submission).

**Figure:**
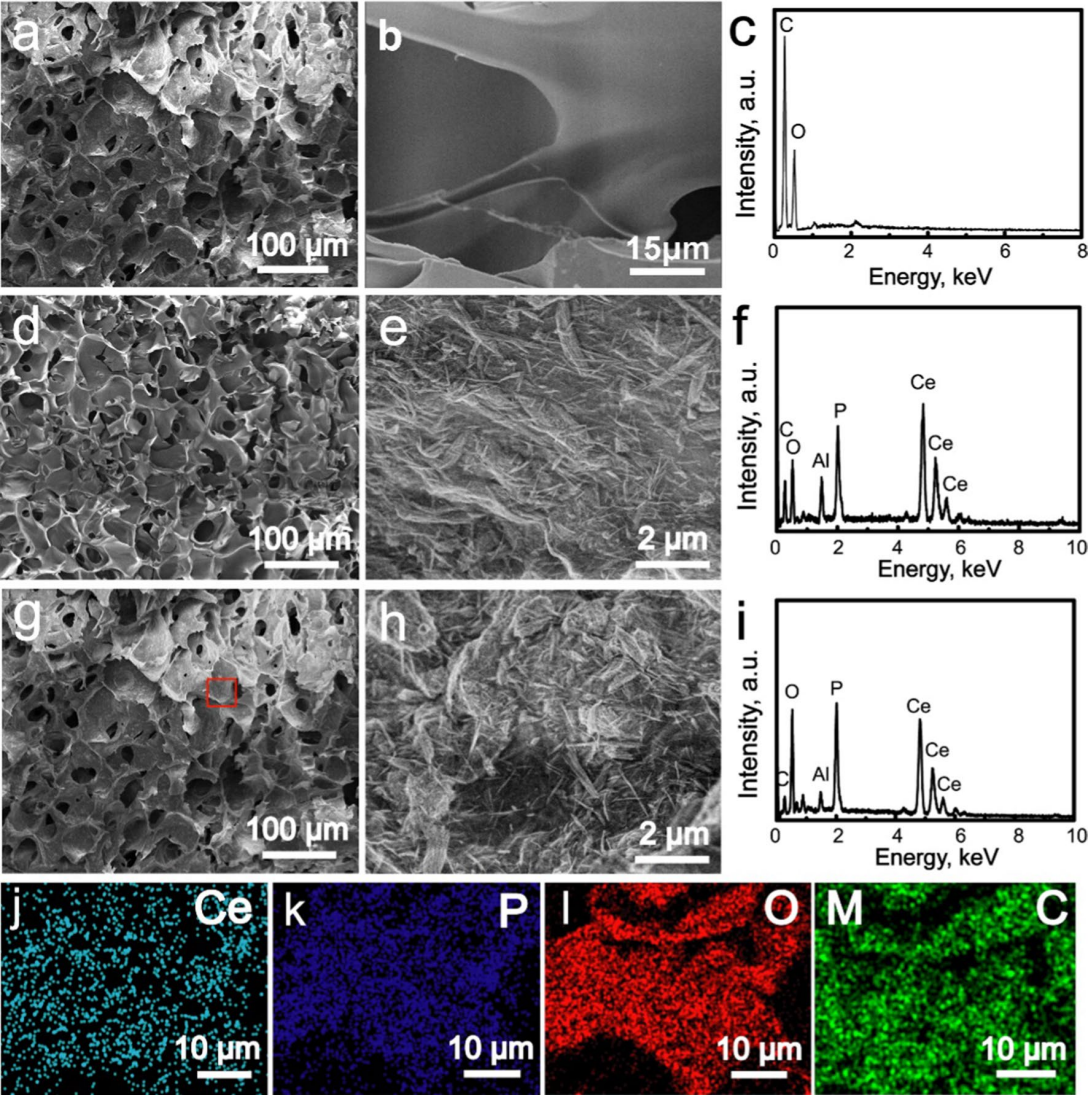
**Fig. 2** **a**, **b** The temperature changes after exposure to NIR radiation. **c**, **d** Fluorescence detection on nude mice after NIR laser irradiation by IVIS Lumina K Series III and fluorescence intensity of the CePO_4_/CS/GO group was significantly lower than the blank, CS and CePO_4_/CS groups. **e**, **f** Optical picture of tumors in nude mice, and quantitative analysis of tumor volume. **g** Histomorphological observation of tumors. Tunel represented apoptosis (blue: nucleus, red: apoptosis), and Caspase-3 represented the most important terminal cleavage enzyme in the process of apoptosis

**Corrected Fig.**2:

**Fig. 2:**
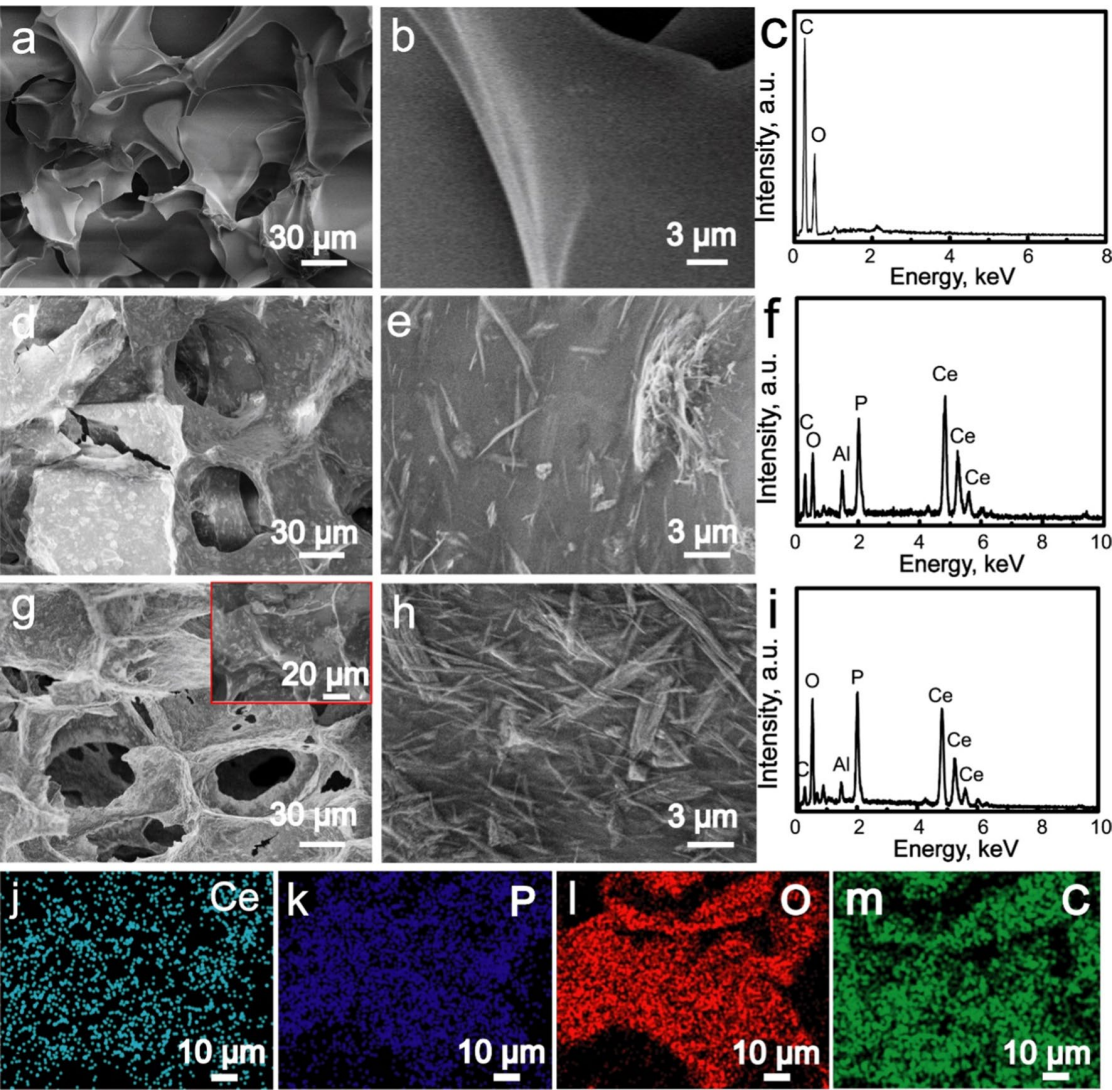
**a**, **b** SEM images and **c** EDS pattern of CS scaffolds; **d**, **e** SEM images and **f** EDS pattern of CePO_4_/CS scaffolds; **g**, **h** SEM images, **i** EDS pattern, **j**–**m** Ce, P, O and C element distribution images of CePO_4_/CS/GO scaffolds which corresponded to the red block in image (**g**)

**Figure 5 before correction** (Fig. 5g was distorted and incorrectly labeled).

**Figure:**
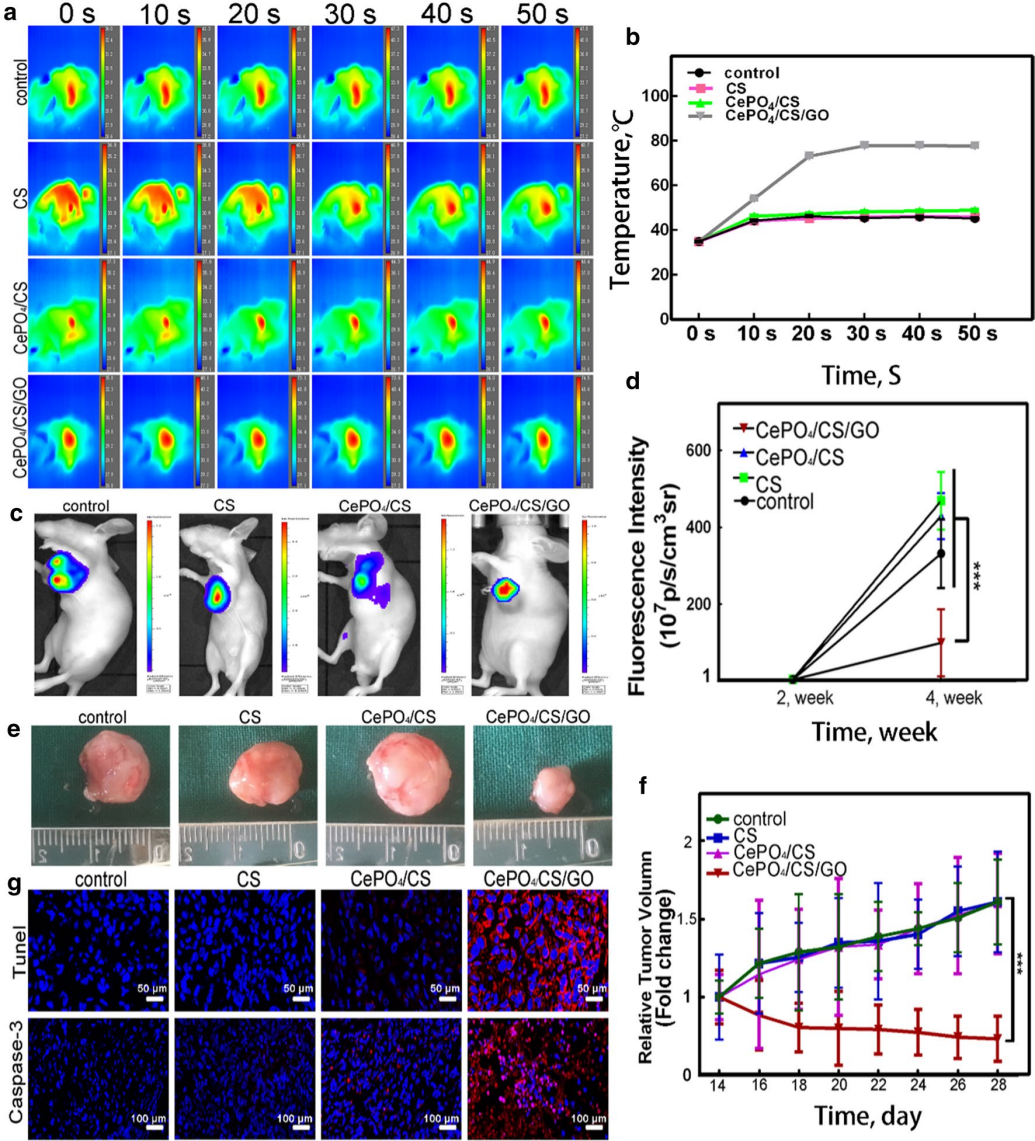
**Fig. 5** **a, b** The temperature changes after exposure to NIR radiation. **c, d** Fluorescence detection on nude mice after NIR laser irradiation by IVIS Lumina K Series III and fluorescence intensity of the CePO_4_/CS/GO group was significantly lower than the blank, CS and CePO_4_/CS groups. **e, f** Optical picture of tumors in nude mice, and quantitative analysis of tumor volume. **g** Histomorphological observation of tumors. Tunel represented apoptosis (blue: nucleus, red: apoptosis), and Caspase-3 represented the most important terminal cleavage enzyme in the process of apoptosis

**Corrected Fig.** 5:

**Fig. 5:**
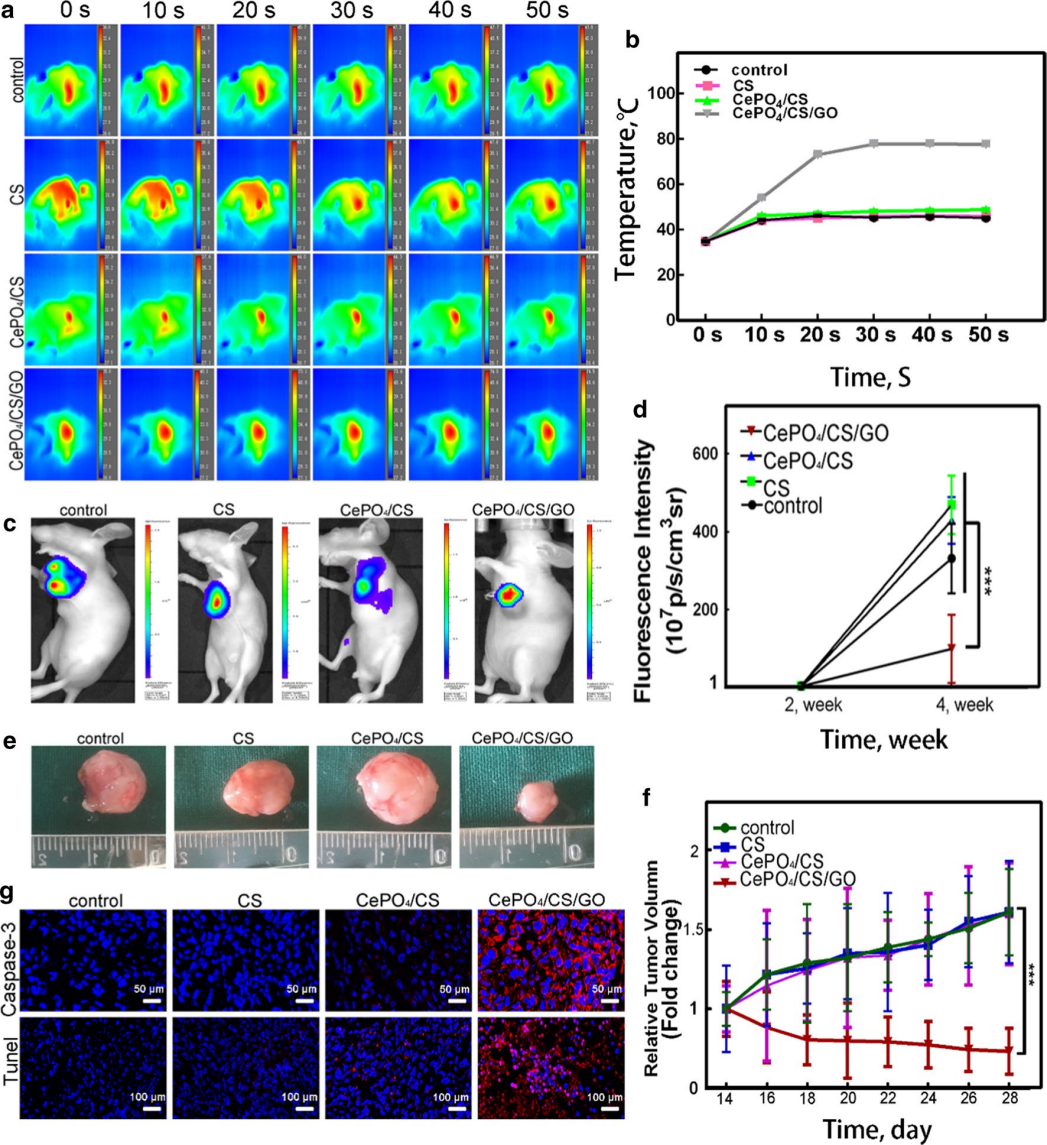
**a, b** The temperature changes after exposure to NIR radiation. **c, d** Fluorescence detection on nude mice after NIR laser irradiation by IVIS Lumina K Series III and fluorescence intensity of the CePO_4_/CS/GO group was significantly lower than the blank, CS and CePO_4_/CS groups. **e, f** Optical picture of tumors in nude mice, and quantitative analysis of tumor volume. **g** Histomorphological observation of tumors. Tunel represented apoptosis (blue: nucleus, red: apoptosis), and Caspase-3 represented the most important terminal cleavage enzyme in the process of apoptosis
