# Supplementary figures and images for: Graphene-modified CePO4 nanorods effectively treat breast cancer-induced bone metastases and regulate macrophage polarization to improve osteo-inductive ability
Source: J Nanobiotechnology. 2021 Jan 7;19:11. doi: 10.1186/s12951-020-00753-9 (PMC7792230; doi:10.1186/s12951-020-00753-9)

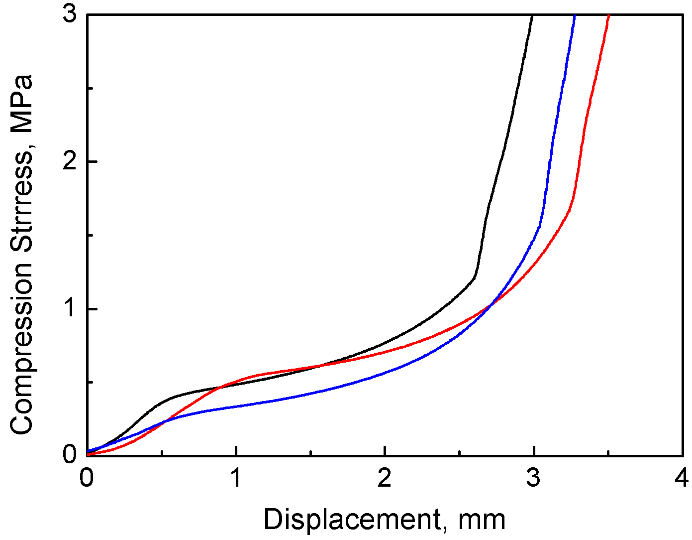


Figure. S1. Compression properties of CePO4/CS/GO scaffolds (*n*=3).

Supplement: Supplementary file 1 — Additional file 1: Figure S1. Compression properties of CePO4/CS/GO scaffolds (n=3). [file 12951_2020_753_MOESM1_ESM.docx]
